# Assessing test–retest reliability of patient-reported outcome measures using intraclass correlation coefficients: recommendations for selecting and documenting the analytical formula

**DOI:** 10.1007/s11136-018-2076-0

**Published:** 2018-12-13

**Authors:** Shanshan Qin, Lauren Nelson, Lori McLeod, Sonya Eremenco, Stephen Joel Coons

**Affiliations:** 1 RTI Health Solutions, 3040 Cornwallis Road, Research Triangle Park, NC 27709 USA; 2 Critical Path Institute, 1730 E River Road, Tucson, AZ 85718 USA

**Keywords:** ICC, Intraclass correlation coefficient, PRO, Patient-reported outcome measures, Test–retest reliability

## Abstract

**Purpose:**

The US Food and Drug Administration (FDA) 2009 guidance for industry on patient-reported outcome (PRO) measures describes how the Agency evaluates the psychometric properties of measures intended to support medical product labeling claims. An important psychometric property is test–retest reliability. The guidance lists intraclass correlation coefficients (ICCs) and the assessment time period as key considerations for test–retest reliability evaluations. However, the guidance does not provide recommendations regarding ICC computation, nor is there consensus within the measurement literature regarding the most appropriate ICC formula for test–retest reliability assessment. This absence of consensus emerged as an issue within Critical Path Institute’s PRO Consortium. The purpose of this project was to generate thoughtful and informed recommendations regarding the most appropriate ICC formula for assessing a PRO measure’s test–retest reliability.

**Methods:**

Literature was reviewed and a preferred ICC formula was proposed. Feedback on the chosen formula was solicited from psychometricians, biostatisticians, regulators, and other scientists who have collaborated on PRO Consortium initiatives.

**Results and conclusions:**

Feedback was carefully considered and, after further deliberation, the proposed ICC formula was confirmed. In conclusion, to assess test–retest reliability for PRO measures, the two-way mixed-effect analysis of variance model with interaction for the absolute agreement between single scores is recommended.

The US Food and Drug Administration (FDA) 2009 guidance for industry on patient-reported outcome (PRO) measures describes how the agency will review and evaluate the development and psychometric properties of measures intended to support medical product labeling claims [1]. Within the psychometric measurement section of the guidance, a key property for review is test–retest reliability, defined as the “stability of scores over time when no change is expected in the concept of interest.” The guidance also lists intraclass correlation coefficients (ICCs) and the time period of assessment as key considerations in FDA review of the test–retest reliability evaluations. While the guidance describes a number of factors to consider when identifying the time period most appropriate for assessments (e.g., variability of the disease state, reference period of the measure), it does not provide specific recommendations regarding the computation of ICCs.

Within the measurement literature, a variety of computational methods have been used to calculate ICCs, a finding that is further complicated by the use of different notation systems for documenting the selected ICC formula [2–7]. The lack of a consistent approach has resulted in confusion regarding which ICC formula is appropriate for assessing test–retest reliability and the inability to compare ICC results across PRO measures when different formulas are used. This absence of consensus regarding the most appropriate ICC formula specific for the assessment of test–retest reliability in PRO measurement and the lack of a uniform naming convention for the ICC formulas emerged as an issue within Critical Path Institute’s (C-Path’s) PRO Consortium [8]. C-Path is an independent, nonprofit organization established in 2005 with public and private support to bring together scientists and others from regulatory agencies, industry, patient groups, and academia to collaborate on improving the medical product development process. C-Path, in cooperation with FDA and the pharmaceutical industry, formed the PRO Consortium in 2008 to facilitate precompetitive collaboration aimed at advancing the assessment of patient-reported and other patient-focused clinical outcomes in drug treatment trials. There was a realization that different, often unidentified, ICC formulas were used by the PRO Consortium’s working groups to evaluate the test–retest reliability of its developmental PRO measures without a clear rationale. This made comparison of test–retest reliability among the measures problematic and, ultimately, complicated regulatory submissions due to the absence of a coherent and consistent approach to ICC formula selection.

To address these issues, the authors reviewed the literature and developed recommendations for the most appropriate ICC formula to fulfill their test–retest reliability objective along with the rationale for the recommendations. The draft of this document was provided to a group of twelve experts including psychometricians, biostatisticians, regulators, and other scientists representing the PRO Consortium, the pharmaceutical industry, clinical research organizations, and consulting firms for review and comment. Feedback was received in written form, followed by discussion with some of the experts for further input and clarification. The authors considered the group’s input in generating the final recommendations presented in this manuscript for selecting the most appropriate ICC formula within the context of assessing the test–retest reliability of PRO measures to support regulatory review.

In the measurement literature, Shrout and Fleiss [5] and McGraw and Wong [6] appear to be the two most cited references for evaluating test–retest reliability. The seminal work of Shrout and Fleiss [5] presented six computational formulas for ICCs. McGraw and Wong [6] expanded the number from 6 to 10 by incorporating more model assumptions, various study designs, and the corresponding analysis of variance (ANOVA) models into the list of considerations for selecting an ICC formula. Because McGraw and Wong [6] offered a more comprehensive treatment of the selection of an ICC formula and a clearer statement of model assumptions, we recommend using their notational system for clarity. However, a key limitation in the general ICC literature is the use of “raters” in the formulas and in the examples, which does not easily translate to the PRO measurement situation where different “time points” rather than different “raters” is the context for the evaluation.

McGraw and Wong present 10 ICC formulas [6, pp 34–35] from which researchers may select based on factors that include the study design (e.g., multiple ratings per subject or multiple subjects per raters), the number of time points, and the intended generalizability of the findings. To assess test–retest reliability for PRO measures, we recommend the two-way mixed-effect ANOVA model with interaction for the absolute agreement between single scores as the preferred ICC formula based on typical study designs (Table 1).

**Table 1:** Two-way mixed-effect analysis of variance (ANOVA) model

Case 3 model of McGraw and Wong [6, p34]:	Two-way mixed model with interaction ${{\varvec{x}}_{{\varvec{i}}{\varvec{j}}}}=\varvec{\mu}+{{\varvec{p}}_{\varvec{i}}}+{{\varvec{t}}_{\varvec{j}}}+{\varvec{p}}{{\varvec{t}}_{{\varvec{i}}{\varvec{j}}}}+{{\varvec{e}}_{{\varvec{i}}{\varvec{j}}}}$, where $\varvec{\mu}$: grand mean ${{\varvec{p}}_{\varvec{i}}}:$ difference due to patient *i* (*i* = 1, …, n), ${{\varvec{p}}_{{\varvec{i}}~}}\sim ~{\mathbf{Normal}}(0,~~\varvec{\sigma}_{{\varvec{p}}}^{2})$ ${{\varvec{t}}_{\varvec{j}}}:$ difference due to time point *j* (*j* = 1, …, k), $\mathop \sum \limits_{{{\varvec{j}}=1}}^{{\varvec{k}}} {{\varvec{t}}_{\varvec{j}}}=0$ ${\varvec{p}}{{\varvec{t}}_{{\varvec{i}}{\varvec{j}}}}:$ interaction between patient *i* and time point *j*, $\mathop \sum \limits_{{{\varvec{j}}=1}}^{{\varvec{k}}} {\varvec{p}}{{\varvec{t}}_{{\varvec{i}}{\varvec{j}}}}=0$ and ${\varvec{p}}{{\varvec{t}}_{{\varvec{i}}{\varvec{j}}}}~\sim ~{\mathbf{Normal}}(0,~~\varvec{\sigma}_{{{\varvec{p}}{\varvec{t}}}}^{2})$ ${{\varvec{e}}_{{\varvec{i}}{\varvec{j}}}}$: random error, ${{\varvec{e}}_{{\varvec{i}}{\varvec{j}}}}~\sim ~{\mathbf{Normal}}(0,~~\varvec{\sigma}_{{\varvec{e}}}^{2})$			

Source of variance		*df*	*MS*	Expected components in *MS*
Between patients		n − 1	*MS*_*P*_	$k\sigma _{p}^{2}+\sigma _{e}^{2}$
Within patients				
Between time points		k − 1	*MS*_*T*_	$n\mathop \sum \limits_{{j=1}}^{k} t_{j}^{2}/(k - 1)+\frac{k}{{k - 1}}\sigma _{{pt}}^{2}+\sigma _{e}^{2}$
Error (p × t)		(n − 1)(k − 1)	*MS*_*E*_	$\frac{k}{{k - 1}}\sigma _{{pt}}^{2}+\sigma _{e}^{2}$
ICC (*A*, 1) of McGraw and Wong [6, p 35] =$\frac{{\varvec{\sigma}_{{\varvec{p}}}^{2} - \varvec{\sigma}_{{{\varvec{p}}{\varvec{t}}}}^{2}/({\varvec{k}} - 1)}}{{\varvec{\sigma}_{{\varvec{p}}}^{2}+\varvec{\sigma}_{{{\varvec{p}}{\varvec{t}}}}^{2}+\varvec{\sigma}_{{\varvec{e}}}^{2}+\mathop \sum \nolimits_{{{\varvec{j}}=1}}^{{\varvec{k}}} {\varvec{t}}_{{\varvec{j}}}^{2}/({\varvec{k}} - 1)}}~=\frac{{{\varvec{M}}{{\varvec{S}}_{\varvec{P}}} - {\varvec{M}}{{\varvec{S}}_{\varvec{E}}}}}{{{\varvec{M}}{{\varvec{S}}_{\varvec{P}}}+({\varvec{k}} - 1){\varvec{M}}{{\varvec{S}}_{\varvec{E}}}+({\varvec{k}}/{\varvec{n}})({\varvec{M}}{{\varvec{S}}_{\varvec{T}}} - {\varvec{M}}{{\varvec{S}}_{\varvec{E}}})}}$				

*A* absolute agreement, *E, e* error, *k* number of time points, *MS* mean squares, *n* number of patients in the test–retest evaluation, *P, p* patients, *T, t* time points

In a typical test–retest assessment with two time points, k is 2 in the above ANOVA model and ICC (A, 1) formula. SAS Proc GLM and Proc Mixed can be used to generate the components needed to compute the intraclass correlation coefficient (ICC). Programming information is available upon request to the corresponding author, and a publicly available macro for computing ICCs in the notational system of Shrout and Fleiss can be found at the SAS website http://support.sas.com/kb/25/031.html

The confidence interval formula of ICC (A, 1) for case 3 model of McGraw and Wong [6] can be found on page 41 of the original paper

This recommendation is based on the following considerations:

1. The two-way model is recommended over the one-way model because time is a design factor in a typical test–retest assessment and the two time points are not interchangeable (i.e., the chronology is important to detect systematic differences such as learning). An ICC computed using the one-way model would underestimate the reliability due to not partitioning the within-patient variability into the time variability and the error term.
2. A mixed-effect model is recommended over a random effect model because, in the former, test and retest time points are prespecified and identical across all study subjects rather than being randomly selected from the population of all possible pairs of time points. In this case, the time effect is considered as fixed.
3. The time-by-subject interaction is assumed to be included in the error term because the interaction cannot be estimated for situations with only one measurement per subject collected at each time point.
4. Absolute agreement is recommended over consistency because subjects are assumed to be stable for the construct of interest across the two time points. Therefore, the systematic differences in the individual scores on the PRO measure over time are of interest.

There are situations where alternative models are more appropriate, however. For example, when the time points for the test–retest assessment could be considered randomly selected (e.g., any two assessments from a number of assessments in the study) in order to generalize the test–retest reliability of the measure beyond the stated context of use, the use of a two-way random effect model is reasonable. In addition, the proposed ICC formula assumes the use of the same mode of data collection for all time points assessed; an alternative ICC formula may be appropriate for the assessment of measurement equivalence between different modes of data collection of the same PRO measure [9].

Note that the ICC (A,1) values remain the same no matter which two-way ANOVA model is constructed. However, we advocate for the articulation of model choice because of the different conceptual considerations being implied. There are many such statistical models where model assumption and interpretation are conceptually different, but some statistics or test results could be the same (e.g., univariate repeated measures ANOVA vs. multivariate ANOVA, and Rasch model vs. 1-parameter logistic item response theory model). We believe that making a clear distinction among models conceptually is important as the chosen model informs the context and the study design. As Schuck [10] noted, “The most important conclusion of the foregoing discussion is not to report ‘the’ ICC, but to describe which ICC has been used, and for what reason.” Whatever the circumstances, we recommend the inclusion of details that describe the exact model used to estimate the ICC and the rationale for the choice. To facilitate the selection of ICC formulas for different study designs (particularly those that are not typical for test–retest reliability evaluation), a decision tree adapted from McGraw and Wong’s published decision tree is provided (Fig. 1).

**Fig. 1:**
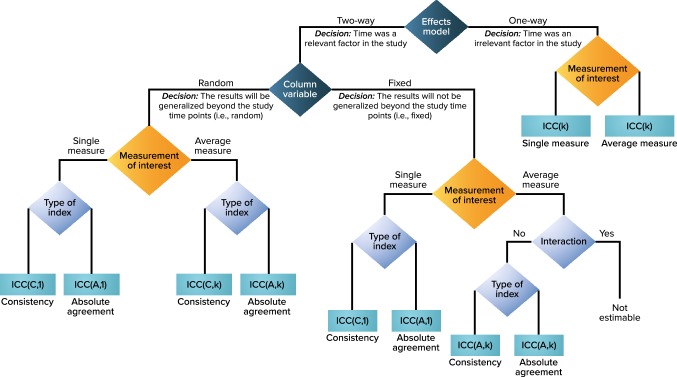
Test–retest ANOVA Model and ICC Type Decision Flowchart (adapted with permission from [6], American Psychological Association). *A* absolute agreement, *ANOVA* analysis of variance, *C* consistency, *ICC* intraclass correlation coefficient, *k* average of k independent measurements

Test–retest ICC values obtained from specific data sets are only point estimates of the true ICC, and they are affected by sample size, data variability, measurement error, and correlation strength as well as by systematic difference between time points [2, 4, 6, 11]. In addition to observed ICC values, we recommend always reporting the corresponding confidence intervals to evaluate the precision of the estimate [6, 12, 13]. When unexpected ICC values occur, additional investigations should be conducted to identify potential reasons for the unexpected values. Investigations to consider include the generation of scatter plots and ANOVA tables and/or conduct of additional correlation assessments, t-tests, or subgroup analyses.

Finally, as ratios of variance components, ICCs of the same model and sample that are calculated using different programming software may vary slightly due to differences in the handling of missing values and the estimation algorithms for variance parameters. Also, due to the fact that between-subject variability is incorporated as part of the ICC ratio, an ICC value is not independent of the study design or specific sample utilized [2]. Low ICC values may be indicative of issues with the study design rather than with the measurement properties of the assessment tool being evaluated. The study population may be restricted to a very narrow subset of scores on the PRO measure’s full score range, for example, and thereby restrict between-subject variability. For these reasons and many others, ICC values should be considered as only a single part of the total evidence needed to support the reproducibility of a PRO measure.
